# The sensitivity and specificity of wideband absorbance measure in identifying pathologic middle ears in adults living with HIV

**DOI:** 10.4102/sajcd.v68i1.820

**Published:** 2021-09-30

**Authors:** Ben Sebothoma, Katijah Khoza-Shangase, Duane Mol, Dipuo Masege

**Affiliations:** 1 Department of Speech Pathology and Audiology, Faculty of Humanities, University of the Witwatersrand, Johannesburg, South Africa; 2 Department of Otorhinolaryngology – Head and Neck Surgery, The Ear and Eye Clinic, Alberton, South Africa; 3 Department of Otorhinolaryngology – Head and Neck Surgery, Faculty of Health Science, University of the Witwatersrand, Johannesburg, South Africa

**Keywords:** adults, HIV, middle ear pathologies, sensitivity, specificity, wideband absorbance at TPP

## Abstract

**Background:**

Limited research exists on the sensitivity and specificity of wideband acoustic immittance (WAI) in adults living with human immunodeficiency virus (HIV). This study forms part of the bigger study titled ‘wideband acoustic immittance in adults living with HIV’.

**Objectives:**

To determine the sensitivity and specificity of the wideband absorbance measure at tympanic peak pressure (TPP), as a screening tool for detecting middle ear pathologies in adults living with HIV.

**Method:**

A prospective nonexperimental study comprising 99 adults living with HIV was performed. All participants underwent a basic audiological test battery which included case history, video otoscopy, tympanometry, wideband absorbance at TPP and pure tone audiometry. Middle ear pathologies were established by two otorhinolaryngologists using asynchronous video otoscopic images analysis. The outcomes of the otorhinolaryngologists served as the gold standard against which the wideband absorbance at TPP and tympanometry were measured. The receiver operating characteristics (ROC) curve was calculated.

**Results:**

ROC revealed the sensitivity of wideband absorbance at TPP to be higher in low to mid frequencies, but significantly lower in frequencies above 971.53 Hz. The sensitivity of tympanometry was lower. However, there was no difference between the specificity of wideband absorbance at TPP and tympanometry, indicating that when there are no pathologies, tympanometry is equally accurate.

**Conclusion:**

The current findings reveal that wideband absorbance at TPP can distinguish middle ear pathologies better than the tympanometry. Incorporating wideband absorbance at TPP in clinical practice may improve early identification and intervention of middle ear pathologies.

## Introduction

Although there have been global efforts to eradicate HIV using antiretroviral therapy (ART) (Mwaba, Ngoma, Kusanthan, & Menon, 2015) and other preventative measures (Williams et al., 2006), there has been a consistent pattern in the literature linking HIV with middle ear pathologies (Sebothoma & Khoza-Shangase, 2020; Tshifularo, Govender, & Monama, 2013; Van der Westhuizen, Swanepoel, Heinze, & Hofmeyr, 2013). Unfortunately, the persistence of middle ear pathologies in individuals living with HIV seems to occur despite the efficacy of ART. Therefore, there is a need for early identification measures for the prevention or reduction of the sequelae of middle ear pathologies associated with HIV.

Several middle ear pathologies have been reported in the literature. Commonly reported middle ear pathologies in adults living with HIV range from acute otitis media (AOM), recurrent otitis media (ROM), otitis media with effusion (OME) to chronic suppurative otitis media (CSOM) (Sebothoma & Khoza-Shangase, 2018; Tshifularo et al., 2013). If middle ear pathologies are left undetected and untreated or detected late, they can invade other auditory compartments (e.g. inner ear), cause permanent auditory damage (Kolo, Salisu, Yaro, & Nwaorgu, 2012) and life-threatening conditions such as intracranial complications (Ibrahim, Cheang, & Nunez, 2010).

Consequences of hearing impairment have been documented (Anderson, Paebery-Clark, White-Schwoch, Drehobl, & Kraus, 2013), which show perception of speech challenges in background noise that may continue despite amplification. Consequently, this may affect overall communication and contribute to the development of psychosocial problems leading to a reduced quality of life (Ciorba, Berto, Borgonzoni, Grasso, & Martini, 2007). Permanent hearing loss may also have financial implications for individuals and the country (Kuchkin, 2018). A systematic review indicates that permanent hearing loss is associated with billions of dollar in excess cost in the United states of America (Huddle et al., 2017). Early identification of middle ear pathologies is therefore crucial.

While less severe forms of middle ear pathologies such as AOM can be treated effectively with simple antibiotics at primary health care (PHC) centres, chronic middle ear pathologies are often difficult to treat. These pathologies may require specialised services that are commonly found in tertiary hospitals or in the private healthcare sector, especially within the South African context (Fagan & Jacobs, 2009). Access to such services is therefore limited because of capacity versus demand challenges (Khoza-Shangase, 2021). Consequently, individuals may opt for ‘self-treatment’ using various hazardous methods (Joubert, Sebothoma, & Kgare, 2017) that are known to cause or exacerbate auditory conditions. Simple and affordable measures that can accurately identify early signs of middle ear pathologies are crucial.

Tympanometry with single probe tone frequency (e.g. 226 Hz) remains a routine audiological measure for assessing middle ear pathologies (British Society of Audiology [BSA], 2013; MacDonald & Green, 2001; Sebothoma & Khoza-Shangase, 2021) because it is easy to administer and produces objective results. However, several studies have demonstrated that tympanometry with a single probe tone is most likely to miss many middle ear pathologies or inaccurately detect them (Kaf, 2011; Sebothoma & Khoza-Shangase, 2018; Shahnaz, Bork, Polka, Longridge, & Bell, 2009). Subjective clinical examination technique are reported to have higher sensitivity and specificity than the standard tympanometry with a single probe tone frequency (Khater, Fahmy, Shahat, & Khater, 2015), and these measures require specialised skills currently only possessed by physicians such as otorhinolaryngologists.

With the continued shortage of otorhinolaryngologists in developing countries such as South Africa (Fagan & Jacobs, 2009), the use of subjective clinical examination technique can delay the diagnosis of middle ear pathologies and render early identification and management impossible. Therefore, there is a need for measures that can combine simplicity, accessibility and high sensitivity and specificity in these contexts.

Wideband acoustic immittance (WAI) has emerged as a potential measure that can accurately identify pathologic middle ears (Hunter & Shahnaz, 2014; Sanford, Hunter, Feeney, & Nakajima, 2013). All these studies have reported that WAI is superior in identifying middle ear pathologies than the standard tympanometry with single probe tone. Terzi et al. (2015) reported a sensitivity of 100% and 94% when wideband average (0.375 KHz – 2.000 KHz) is used. Other studies have reported that WAI can also predict conductive hearing loss (CHL) (Keefe, Sanford, Ellison, Fitzpatrick, & Gorga, 2012; Prieve, Feeney, Stenfelt, & Shahnaz, 2013). Keefe et al. (2012) found that WAI can predict the presence and magnitude of CHL of air bone gap (ABG) that ≥ 20 dB at frequencies above 0.7 KHz.

The superiority of WAI over standard tympanometry with single probe tone is attributed to its clinical parameters. Wideband acoustic immittance uses stimuli with broad spectrum such as clicks assessed over a wide range of frequencies, typically from 220 Hz to 11 000 Hz to measure middle ear functioning (Margolis, Saly & Keefe, 1999). Unlike tympanometry with single probe tone, WAI is also shown to be able to measure the resonance frequency (RF) of the middle ear system. This renders the measure more sensitive and specific and therefore arguably appropriate for early detection of middle ear pathologies in the population most at risk of these conditions. However, there is limited research on the sensitivity and specificity of WAI in identifying middle ear pathologies in adults living with HIV. Hence, the importance of this study, which intends to contribute to the existing body of knowledge regarding the sensitivity and specificity of WAI. This is crucial in South Africa where there is a high burden of HIV disease. Therefore, the aim of this study was to determine the sensitivity and specificity of the wideband absorbance measure at tympanic peak pressure (TPP), as a screening tool for detecting middle ear pathologies in adults living with HIV.

## Methods

### Research design

This study employed a prospective, non-experimental design (Leedy & Ormrod, 2013). Data for this study was quantitative and variables of interests were described without any manipulation (Irwin Pannbacker, & Lass, 2014).

### Participants

Non-probability purposive sampling was employed to select and recruit potential participants (Leedy & Ormrod, 2013). Purposive sampling was deemed appropriate as it allows the researcher to carefully select participants who exhibit the characteristics that are important and useful for the study (Leedy & Ormrod, 2013). In this study, for example, the inclusion criteria were that participants had to be adults diagnosed with HIV. Several advantages of purposive sampling are documented in the literature. Purposive sampling is a more convenient and cost-effective method to use than other sampling methods. Participants were available at the HIV clinic and could be easily invited to participate in the study. The disadvantage was that non-probability sampling methods do not allow the researcher to generalise the findings to a larger population (Leedy & Ormrod, 2013). Participants were adults aged 18 years and older diagnosed with HIV, attending the NMC. Those who did not have otorrhoea on the day of testing, were invited, agreed and signed an informed consent form.

All participants were black Africans with similar socioeconomic backgrounds. This selection was performed because research has indicated that people from different ethnic groups (Shahnaz & Bork, 2006) and socioeconomic groups (Siddartha, Bhat, Bhandary, Shenoy, & Rashmi, 2012) may have slightly different middle ear function.

### Research context

This study was conducted in one of the HIV clinics in the Limpopo province in South Africa. The clinic established an audiology clinic, which offers various audiological services. The clinic is one of the few in the country to have obtained an immittance machine, which can be used for wideband absorbance measurements. Therefore, the choice of the study site was based on the availability of equipment and population being served at the clinic.

### Procedure

All participants underwent a basic audiological test battery, which included case history information using a self-developed abstraction sheet, video otoscopy using Aurica otocam 300. Tympanometry with 226 Hz probe tone and wideband absorbance at TPP were performed using a Titan Interacoustic, Denmark, version 3.3. Pure tone audiometry was conducted using an AD 629 diagnostic Interacoustics audiometer with Sennheiser HA 200 supra-aural headphones and Radio Ear B-71 bone conductor. All these audiological equipment were calibrated 6 months prior the commencement of the study (Jnaurary 2018). Speech reception thresholds (SRTs) were performed using the South African Spodaic (SAS) wordlist (Hanekom, Soer, & Pottas, 2015). The Titan machine was connected to the Acer laptop via a USB cable.

An appropriate probe nub that provided a better hermetic seal was used to measure tympanometry using a 226 Hz probe tone and wideband absorbance at TPP. A single low frequency probe tone of 226 Hz at an intensity of 85 dB sound pressure level (SPL) was presented into the ear. To generate tympanograms, pressure was swept from +200 to –400 daPa. Acoustic compliance, pressure (daPa) and ear canal volumes (ECVs) were analysed to determine the absence of a type A tympanogram or the presence of type As, Ad, B or C indicative of middle ear pathologies. Normative values that were used to determine the type of tympanogram were those of the BSA (2013). Values that were within the normative range were coded as 1, indicating the absence of middle ear pathology; while values that were out of the normative range were coded 2, indicating the presence of middle ear pathology.

Wideband absorbance at TPP was conducted by changing the function on the Titan equipment. Wideband absorbance at TPP is conducted by varying pressure in the external auditory meatus from +200 to –300 daPa at the rate of 200 daPa/s. Wideband absorbance measure was conducted at TPP because the tympanic membrane is at maximal mobility during TPP (Feeney & Sanford, 2012). Data were collected at 107 frequencies from 0.226 kHz to 8 kHz. However, frequencies were reduced to 16 at 1/6th octave for statistical analysis purposes.

The researchers used pure tone thresholds to determine the hearing sensitivity of each participant. Air and bone conduction thresholds were measured across 250 Hz – 8000 Hz and 250 Hz – 4000 Hz frequencies, respectively. An ABG of ≥ 10 dB was used to determine the presence of middle ear pathology (e.g. CHL or mixed hearing loss) (Kramer & Brown, 2019)

All video otoscopic images were sent to two otorhinolaryngologists using asynchronous (save and forward) telepractice techniques (Biagio, Swanepoel, Laurent, & Lundberg, 2014). The roles of the otorhinolaryngologists were to analyse and make diagnoses of middle ear pathologies based on video otoscopic images. These results served as the gold standard against which to measure the sensitivity and specificity of the tympanometry with 226 Hz probe tone and wideband absorbance at TPP. The motivation for utilising video otoscopic analysis through asynchronous telepractice method as a gold standard was based on clinical practice in South Africa, where the diagnosis for middle ear diseases made by otorhinolaryngologists is considered to be the gold standard (Biagio, Swanepoel, Adeyemo, Hall, & Vinck, 2013). The diagnoses of middle ear pathologies that were performed through the analysis of asynchronous video otoscopic images by the otorhinolaryngologists were found to be similar (*k* = 0.7) to those made via otomicroscopy (Biagio et al., 2014). As a result of the scarcity of otorhinolaryngologists in South Africa, a more convenient method, with a similar level of accuracy was chosen to serve as a gold standard. Otorhinolaryngologists in this study were blinded on the demographic characteristics of the participants, recording and interpretation of the diagnoses.

### Reliability and validity

Test–retest reliability was achieved by conducting a pilot study prior to the main study (Satake, 2015). This pilot study was conducted with five adults who met the inclusion criteria of the main study (Sebothoma & Khoza-Shangase, 2018). All the audiological tests were performed by the first author who is also an experienced clinical audiologist. Each participant was tested individually in a sound treated room in the audiology department at NMC. Calibration of all equipment was carried out before the commencement of the study, in line with the American National Standards Institute (ANSI, 2004). Daily biological calibration was also conducted everyday before testing participants (Wilber & Burkard, 2015).

### Data analysis

Statistical analysis was performed using Statistical Package for Social Science (SPSS) version 24 (Chicago, IL). Descriptive and inferential statistics were employed. The otorhinolaryngologists used a qualitative method to analyse the structure, thickness, colour and position of the tympanic membrane to make diagnoses of any middle ear pathology (Biagio et al., 2013). For the purpose of this study, middle ear pathologies were not pre-defined; instead, were diagnosed by the otorhinolaryngologists using asynchronous video otoscopic images. Cohen’s Kappa was used to evaluate the agreement diagnosis between the two otorhinolaryngologists (Sebothoma & Khoza-Shangase, 2018). The overall test performance of wideband absorbance at TPP and tympanometry with 226 Hz probe tone was evaluated by using receiver operating characteristics (ROC). The (AUROC) curve and 95% confidence interval (CI) were calculated automatically on SPSS version 24. Chi squared was also used to determine the association between the diagnosis made between the two otorhinolaryngologists and hearing loss with conductive component.

### Ethical considerations

This research project was approved by the Human Research Ethics Committee (HREC) (Medical) (M160663) of the University of the Witwatersrand, Johannesburg. Permission was granted by the provincial Department of Health (the Limpopo province) and by the chief executive officer (CEO) of NMC. All participants gave written consent before commencing with the tests.

## Results

Of the 198 ears included in the study, the two otorhinolaryngologists initially excluded 25 ears (13%) of the video otoscopic images because of poor visualisation of the tympanic membrane and wax. Of the 173 ears, Cohen’s Kappa revealed a strong agreement between the diagnoses made by two otorhinolaryngologists (*k* = 0.7). Another 12 ears (7%) were excluded from the study because the two otorhinolaryngologists were not in agreement with the diagnoses of the middle ear pathologies in these patients. Therefore, a total of 161 ears were finally included in the study, 17 had abnormalities (11%) and 144 were normal (89%). Middle ear pathologies that were diagnosed by the otorhinolaryngologists during analysis included OME (*n* = 5), retracted tympanic membrane (*n* = 4), tympanic membrane perforation (*n* = 3), CSOM (*n* = 2), tympanosclerosis (*n* = 1), tympanic membrane scarring (*n* = 1) and otitis externa (*n* = 1).

Table 1 indicates the diagnoses made by each otorhinolaryngologist. A total of 161 ears (87 adults) were finally analysed and diagnosed by the two otorhinolaryngologists.

**TABLE 1:** Summary of video otoscopic diagnoses.

Otorhinolaryngologist	Normal ears		Abnormal ears	
	*n*	%	*n*	%
Otorhinolaryngologist 1	158	91	15	9
Otorhinolaryngologist 2	150	87	23	13

Table 2 and Table 3 summarises the results indicated in the area under the curve that was used to determine the specific cut-off values to determine the sensitivity and specificity of wideband absorbance at TPP, tympanometry with 226 Hz probe tone and pure tone audiometry. Table 4 classifies the sensitivity and specificity of wideband absorbance measure in identifying middle ear pathologies in different frequencies. As the purpose of the study was to determine whether wideband absorbance at TPP has high sensitivity and specificity for identifying middle ear pathology, the criterion used to select cut-off values involves the use of the ROC curve to select the point where the product of the two indices (sensitivity and specificity is maximum). These values were then used to determine the sensitivity and specificity of wideband absorbance at TPP. The procedure for determining the sensitivity and specificity of tympanometry using a 226 Hz probe tone and pure tone audiometry was similar.

**TABLE 2:** Area under the curve for wideband absorbance measure.

Frequency	AUROC	95%	CI
226.00	0.591	0.452	0.731
363.91	0.600	0.473	0.726
514.65	0.629	0.512	0.747
667.42	0.641	0.520	0.761
793.70	0.631	0.502	0.759
971.53	0.581	0.432	0.730
1155.35	0.452	0.288	0.616
1414.21	0.446	0.293	0.599
1781.80	0.493	0.339	0.647
2058.60	0.448	0.284	0.612
2519.84	0.441	0.282	0.599
3084.42	0.465	0.305	0.625
3775.50	0.416	0.271	0.560
4495.80	0.438	0.309	0.567
5495.80	0.417	0.251	0.584
6535.60	0.346	0.211	0.481

AUROC, area under the ROC; CI, confidence interval.

**TABLE 3:** Area under the curve for tympanometry with 226 Hz and pure tone audiometry.

Test	AUROC	9.5%	CI
Tympanometry with 226 Hz probe tone	0.61	0.432	0.788
Pure tone audiometry	0.560	0.406	0.715

AUROC, area under the ROC; CI, confidence interval.

**TABLE 4:** The sensitivity and specificity of wideband absorbance measurements.

Frequency	Cut-off value	Sensitivity (%)	Specificity (%)
226.00	0.135	59	65
363.91	0.280	77	41
514.65	0.435	71	46
667.42	0.704	82	39
793.70	0.641	65	60
971.53	0.727	71	53
1155.35	0.614	30	83
1414.21	0.592	23.5	85
1781.80	0.556	23.5	81
2058.60	0.497	23.5	87
2519.84	0.541	23.5	82
3084.42	0.346	23.5	90
3775.50	0.249	18	83
4495.80	0.524	88	20
5495.80	0.139	23.5	88
6535.60	0.079	12	90

The sensitivity of wideband absorbance at TPP was higher (59% – 82%) in low to mid frequencies (226 Hz – 971.43 Hz), but significantly lower (23.5%) at frequencies above 971.43 Hz (see Table 3). The Mann–Whitney U test (Nachar, 2008) revealed a statistically significant difference (*p* < 0.05) between the sensitivity of wideband absorbance at TPP and the sensitivity of tympanometry with 226 Hz prone tone, especially in low to mid frequencies. The specificity of wideband absorbance at TPP was generally high across frequencies. There was no statistical difference between the specificity of wideband absorbance at TPP and the specificity of tympanometry using a 226 Hz probe (93.8%).

The sensitivity and specificity of pure tone audiometry in detecting middle ear pathologies were also established. The sensitivity of pure tone audiometry using the ABG as an indicator was 17.6%, while the specificity of ABG was 44%. The Chi-squared analysis revealed that there was a statistically significant association (*p* = 0.62) between middle ear pathologies as identified by the otorhinolaryngologists analysis and actual diagnosed conductive pathology (conductive and mixed hearing loss).

## Discussion

This study evaluated the sensitivity and specificity of wideband absorbance at TPP compared with the standard tympanometry with 226 Hz probe tone in adults living with HIV. To the knowledge of the authors these are the first results of wideband absorbance at TPP in adults living with HIV to date. Although various types of middle ear pathologies may differentially influence the mass and compliance component of the middle ear system and may potentially be a major factor contributing to the difference in sensitivity and specificity, the prevalence of specific middle ear pathologies in this study was significantly low (*n* = 13). As a result, the sensitivity and specificity of wideband absorbance at TPP could not be linked to specific middle ear pathologies. Instead, the researchers ended up with two categories, indicating ‘presence’ or ‘absence’ of middle ear pathologies. Further studies are therefore required to investigate the sensitivity and specificity of wideband absorbance at TPP in adults living with HIV for specific middle ear pathologies.

The sensitivity of wideband absorbance at TPP was generally higher than the sensitivity of tympanometry with 226 Hz probe tone and pure tone audiometry in detecting middle ear pathologies. However, the specificity of wideband absorbance at TPP was comparable to the specificity of tympanometry with 226 Hz probe tone. The findings of this study are consistent with previous studies, which indicated that WAI absorbance or reflectance is superior in detecting middle ear pathologies compared with tympanometry with single probe tone (Terzi et al., 2015). Shahnaz et al. (2009) observed that tympanometry with a 226 Hz probe tone is less sensitive to middle ear pathologies, which do not severely affect the mechanical properties of the tympanic membrane such as otosclerosis. Often these pathologies alter the RF but may retain the normal functioning of the tympanic membrane (Iacovou, Vlastarakos, Ferekidis, & Nikolopoulos, 2013).

While the sensitivity and specificity of wideband absorbance at TPP was generally higher in this study; it is lower compared with previous studies (Beers, Shahnaz, Westerberg, & Kozak, 2010; Terzi et al., 2015). This variation may be attributed to various factors. Firstly, the AUROC values were obtained, which were used to generate cut-off values that were generally lower in this study compared with previous studies. For example, a minimum AUROC of 0.56 and maximum of 0.98 across a frequency spectrum (0.25 Hz – 8.00 KHz) was found by Terzi et al. (2015), which indicate a good diagnostic test in distinguishing pathologic ears from non-pathologic ones. However, in this study, AUROC values of 0.41–0.64 were obtained across the frequency spectrum (0.25 Hz – 8.00 KHz). These AUROC values have facilitated the selection of maximum sensitivity and specificity.

The reasons for obtaining such small AUROC values in this study are not clear. Perhaps using the diagnoses and analyses of otorhinolaryngologist as a gold standard may have been a confounding factor. Otorhinolaryngologists used video otoscopic images to analyse the structure and colour of the tympanic membrane to make the appropriate diagnoses. However, wideband absorbance at TPP as part of the acoustic immittance relies on the mechanical aspect of middle ear system (Beers et al., 2010). Literature suggests that there are certain middle ear pathologies that do not affect mechanical aspects of the middle ear system, and therefore acoustic immittance measures may miss these pathologies (Kaf, 2011; Sebothoma & Khoza-Shangase, 2018). Despite this limitation, results have still indicated wideband absorbance at TPP to be a better measure of detecting middle ear pathologies than the standard tympanometry using a 226 Hz probe tone.

Research suggests that there is an association between HIV and middle ear pathologies (Sebothoma & Khoza-Shangase, 2018). Unfortunately, these pathologies persist despite the effectiveness of ART treatment. Given the fact that HIV continues to increase, especially in developing countries (Joint United Nations Programme on HIV/AIDS [UNAIDS], 2018) combined with shortages of health professionals (Mulwafu, Ensink, Kuper, & Fagan, 2017), there is a need to use measures that have high sensitivity and specificity in identifying middle ear pathologies. Wideband absorbance at TPP offers the possibility of accurately identifying pathologic middle ears that could not be identified through the use of tympanometry with 226 Hz probe tone, thereby facilitating early intervention.

These findings support evidence suggesting that wideband absorbance measures can improve early identification of middle ear pathologies (Terzi et al., 2015), especially in individuals who are prone to infection. However, the pattern of sensitivities across frequencies in this study was different from other studies (Beers et al., 2010; Terzi et al., 2015). Although the RF in this study was not calculated, the current findings suggest that wideband absorbance at TPP alone may also miss pathologies affecting the RF, which sometimes can be detectable above 971.43 Hz (Özgür et al., 2016). Therefore, wideband absorbance at TPP may need to be used in conjunction with other measures in order to improve early identification in adults living with HIV. Therefore, further studies are needed in this area.

### Clinical implications

The literature suggests that individuals living with HIV are at increased risk of middle ear pathologies compared with HIV negative control group (Sebothoma & Khoza-Shangase, 2018; Tshifularo et al., 2013). Middle ear pathologies of these individuals vary depending on the number of CD4 cells in the body (Obasineke, Amdi, Ibekwe, Ezeanolue, & Ogisi, 2014; Van der Westhuizen et al., 2013). Some of these pathologies may be missed when using tympanometry using a 226 Hz probe tone. Sebothoma and Khoza-Shangase (2018) observed that the use of tympanometry using a 226 Hz probe tone in identifying pathologic middle ears is ineffective in individuals living with HIV. Despite the difference between the finding of this study and previous studies the recommendation is still made that wideband absorbance at TPP may be useful in clinical practice. Audiologists may be able to identify early signs of middle ear pathologies and offer opportunities for timeous interventions.

Some middle ear pathologies can be successfully treated at PHC centres with antibiotics. However, severe middle ear pathologies may require specialised services, which are limited in developing countries (Fagan & Jacobs, 2009). The utility of WAI in clinical practice may potentially improve early identification and reduce these challenges. With the current proposed model of service delivery in resource-constrained environments, which involves the use of specialised technology (Swanepoel & Clark, 2018), wideband absorbance measure can be embedded in these programmes, increase access to hearing healthcare and thereby reduce costs associated with traveling long distances for treatment. Although there is a promising prospect of using WAI, especially for the identification of middle ear pathology in developing countries and can be carried out without otorhinolaryngologists and audiologists, it may not be considered because of its expense. While testing may take a couple of seconds to complete, trained audiologists are needed to conduct these tests, and the equipment for measuring wideband absorbance measure is considerably more expensive and more sophisticated to operate compared with conventional screening tympanometry. It is not yet known whether paraprofessionals can effectively conduct and interpret wideband absorbance measures.

Much work still needs to be carried out. Increased sample sizes in WAI studies may enable generalisability. Furthermore, studies on WAI must include an HIV negative control group in order to make a comparison of wideband absorbance patterns in adults living with HIV. As acoustic reflex thresholds were not conducted as part of the middle ear assessment, further studies need to include ART in order to improve the accuracy of identifying middle ear pathologies. Lastly, studies on wideband absorbance measure must also measure ambient pressure (Hunter & Shahnaz, 2014), which provides crucial information about the middle ear mechanics.

## Conclusions

The findings of this study reveal that wideband absorbance at TPP can show middle ear pathologies better than the conventional tympanometry with 226 Hz probe tone. Given the high prevalence of HIV in South Africa, and its continued link to the development of middle ear pathologies, incorporating wideband absorbance measure in clinical practice may improve early identification of middle ear pathologies and offer these individuals the opportunity to receive timeous intervention. However, wideband absorbance measures may need to be used in conjunction with other measures, which are more sensitive to middle ear pathologies that have not yet affected the mechanical properties of the tympanic membrane.
